# Evidence for Weak or Linear Conformity but Not for Hyper-Conformity in an Everyday Social Learning Context

**DOI:** 10.1371/journal.pone.0030970

**Published:** 2012-02-20

**Authors:** Nicolas Claidière, Mark Bowler, Andrew Whiten

**Affiliations:** Centre for Social Learning and Cognitive Evolution, University of St Andrews, St Andrews, Scotland, United Kingdom; Queen Mary, University of London, United Kingdom

## Abstract

Conformity is thought to be an important force in cultural evolution because it has the potential to stabilize cooperation in large groups, potentiate group selection and thus explain uniquely human behaviors. However, the effects of such conformity on cultural and biological evolution will depend much on the way individuals are influenced by the frequency of alternative behavioral options witnessed. Theoretical modeling has suggested that only what we refer to as ‘hyper-conformity’, an exaggerated tendency to perform the most frequent behavior witnessed in other individuals, is able to increase within-group homogeneity and between-group diversity, for instance. Empirically however, few experiments have addressed how the frequency of behavior witnessed affects behavior. Accordingly we performed an experiment to test for the presence of conformity in a natural situation with humans. Visitors to a Zoo exhibit were invited to write or draw answers to questions on A5 cards and potentially win a small prize. We manipulated the proportion of existing writings versus drawings visible to visitors and measured the proportion of written cards submitted. We found a strong and significant effect of the proportion of text displayed on the proportion of text in the answers, thus demonstrating social learning. We show that this effect is approximately linear, with potentially a small, weak-conformist component but no hyper-conformist one. The present experiment therefore provides evidence for linear conformity in humans in a very natural context.

## Introduction

Conformity represents an important aspect of human psychology and has been extensively studied in social psychology since the pioneering work of Asch [1], [2]. Studies in human adults (reviewed by [3]) and children [4], [5], [6] have shown that confronting participants with a majority of individuals behaving in a certain way is often enough to make the participant behave in the same way, even when this is in strong conflict with their personal tendency to behave otherwise. In social psychology, the phenomenon of conformity is well established, notably in situations in which participants are trying to achieve effective action, to build and maintain social interactions and to maintain a positive evaluation of themselves [3], [7].

Building on this work, Boyd and Richerson [8] proposed a more restricted sense of the term conformity, that we here call hyper-conformity (see below and Claidière and Whiten [9] for an explanation of the rationale underlying the terminology used here): this designates an exaggerated tendency to perform the most frequent behavior witnessed in other individuals (see also [10], [11]). For example, an individual seeing that 80% of their community exhibit behavior A rather than B would be hyper-conformist if the probability of their performing behavior A significantly exceeded 0.8. This particular notion of conformity has received considerable theoretical attention [8], [11], [12], [13], [14], [15], [16], [17], [18]. In particular, theoretical and modeling studies have shown that hyper-conformity can increase the behavioral homogeneity within groups, and by doing so can (a) facilitate cumulative cultural evolution [19], on page 145: “If we are right, culture is adaptive because it can do things that genes cannot do for themselves. Simple forms of social learning cut the cost of individual learning by allowing individuals to use environmental cues selectively. If you can easily figure out what to do, do it! But if not, you can fall back on copying what others do. When environments are variable and the learning is difficult or costly, such a system can be a big advantage, and most likely explains the relatively crude systems of social learning commonly found in social animals. Humans have evolved the additional capacity to acquire variant traditions by imitation and teaching, and can accurately, quickly, and selectively acquire the most common variant or the variants used by the successful. When these kinds of social learning biases are combined with occasional adaptive innovations and content biases, the result is the cumulative cultural evolution of complex, socially learned adaptations, adaptations that are far beyond the creative ability of any individual.”), (b) explain the existence of maladaptive cultural behaviors, (c) potentiate group selection and thereby stabilize cooperation in large groups (summarized in [19]). These influential theoretical analyses have suggested that hyper-conformity might be one important factor explaining the divergence between human culture and animal culture. Empirically however, evidence for the existence of hyper-conformity in humans remains limited and controversial [16].

To date, only a handful of experiments have attempted to test for the existence of hyper-conformity in humans. A general approach pioneered by McElreath et al. [20] has been to ask participants to play a virtual game in which they could access various kinds of social information (see also [10], [13], [18], [21]. McElreath et al. [20] found that in practice, most participants did not use social information. Those who did were either unbiased or showed a small hyper-conformist tendency. Later studies have investigated further the different strategies participants use when taking decisions in a complex, temporally variable virtual environment. These studies have revealed important inter-individual differences and the use of complex strategies such as ‘choose the best of two options if the average difference between them is large, hyper-conform to what the majority is doing otherwise’ [18]. Such experiments allow a precise control over the information participants can gather (number of participants, strategy, payoff, etc) through the implementation of complex virtual computer games. The drawback however is that the relationship between the behavior of participants in these experiments and in more natural situations remains in question. Ideally, precisely controlled, virtual experiments should be complemented by more natural observations and experiments assessing the role of conformity in more everyday situations.

Only a few experiments have focused on such non-laboratory situations [16], [22], [23]. Coultas [22], for instance, surreptitiously recorded the tendency of participants to perform a normally rare action (balancing a keyboard cover on a computer) when the proportion of individuals doing that action rather than a more common one (putting the cover next to the computer) was varied. The results of this study suggested that participants will not be hyper-conformist unless the proportion of the majority is very high.

Studies that have focused on a conformity bias in these more natural contexts [16], [22], [23] led Eriksson et al. [16] to conclude that:

“Based on our theoretical arguments and simulations, we do not expect any strong selection pressure for a conformist bias, and we found no satisfactory evidence that such a bias actually exists within human psychology.” (p 21, ‘conformist bias’ sensu ‘hyper-conformity’)

Nevertheless, the broader work cited in our opening paragraph suggests that humans (and possibly animals too, see [24]) - are sensitive to the relative frequencies of alternative behavioral options. Given the limited number and context of experiments in which the frequency of different information has been manipulated, the question of the kind of conformity that humans display remains quite open.

**Figure 1:**
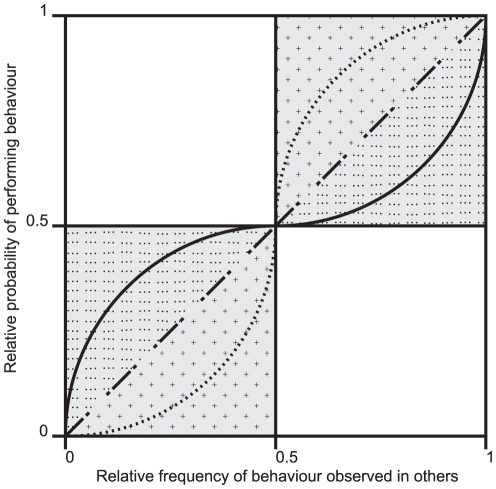
Three different types of conformity. In the shaded domains showing conformity, we distinguish three alternative possibilities: (i) weak-conformity (dotted domain, plain line is a possible example); (ii) linear conformity (dash dotted line); and (iii) hyper-conformity (crossed domain, dotted line is a possible example). The non-shaded domains correspond to anti-conformity, in which individuals adopt the least frequently observed alternative (after [9]).

In this article, we follow the terminology advocated by Claidière and Whiten [9] in a recent review of the literature on conformity. This terminology is meant to limit the confusion that has arisen between the modeling literature and experimental work in social psychology and animal behavior. Accordingly, we will use the term ‘conformity’ to refer to any positive relationship between the frequency of a behavioral variant in a population and its probability of being performed by an individual. The conformity domain is thus represented by the shaded quadrants in Fig. 1. We use the term anti-conformity to refer to any negative relationship between the frequency and the probability to perform a behavior (the unshaded quadrants in the figure).

We also distinguish between three different grades of conformity (Fig. 1):

- Hyper-conformity when the probability that an individual performs the most frequent behavior is greater than the observed frequency of that behavior in others (NB hyperconformity in our terminology has been modelled and defined in [8] as “positive frequency dependent” or “conformist” bias).
- Linear conformity when the probability that an individual performs the most frequent behavior is equal to the observed frequency of that behavior in others (NB modelled and defined in [8] as “unbiased transmission”).
- Weak-conformity when the probability that an individual performs the most frequent behavior is less than the observed frequency of that behavior in others but still larger than the probability of performing the less frequent behavior.

Our experiment aims at establishing the presence or not of conformity in a natural situation with humans as well as distinguishing between the three kinds of conformity represented in Figure 1.

Compared to previous studies, ours takes advantage of a fairly common situation. Amid the exhibits of the ‘Medicine Now’ gallery at the Wellcome Collection (Euston Road, London), visitors are struck by an impressive collection of drawings and writings made by previous visitors and displayed on a large ‘Feedback Wall’. Visitors are encouraged to contribute by leaving their own comments or piece of art on feedback cards, located on tables around the wall. Visitors are asked to pick a word or two from a list of words located on the opposite side of the card (e.g., LOVE, PLACEBO, BRAIN, etc) and then, using crayons provided, draw or write whatever they like on the topic. They can then display their work on the feedback wall.

If there is no effect of the display on the behavior of visitors, what we observe on the wall would be a simple reflection of visitors' natural preference for writing or drawing. According to this first possibility, if the display were covered mostly in text for instance, visitors would still produce the same natural ratio of writings over drawings. A second possibility however, is that the proportion of writings on displays influences the visitors' behavior in such a way that they tend to do more of the most common option. According to this second hypothesis, if the display were covered mostly in text, visitors would produce more writings than when it is covered mostly with drawings. If we were to manipulate the proportion of writings over drawings on display, we would be able to find evidence for or against conformity.

Inspired by the Wellcome Trust display, we devised a similar set-up in the ‘Living Links to Human Evolution’ Research Centre of the University of St Andrews, located in Edinburgh Zoo [25]. All visitors to the Zoo are encouraged to visit this Centre and thousands do so each year. In an experiment described fully below, we presented a display that invited visitors to respond to questions about the Centre on A5 cards and we presented selected contributions on a large board. We manipulated the proportion of writings versus drawings on display on different occasions and measured the proportion of writings in the cards submitted in each period. We were interested in the influence the display could have on the behavior of participants and we predicted one of the forms of conformity that we define above.

More specifically, Richerson and Boyd argue that when individuals have difficulty making decisions because they have limited information, they should tend to be hyper-conformist ([19] p120–124). Hyper-conformity, they claim, is an evolved heuristic that makes us take the most appropriate decision in uncertain situations ([19] p120) and it can operate only when “individuals have difficulty evaluating the costs and benefits of alternative cultural variants” ([19] p206). In our experiment, visitors did not have information regarding the criteria we would use to select responses to win the prize, which could potentially have been based on a complex combination of various factors. For instance, it could have been a weighted combination of the adequacy of the answer, the quality of the drawing or writing, the colors used, and the age and gender of the participant. Given the limited amount of information and the uncertainty regarding the outcome of the selection process, theory predicts visitors should be guided by the display (see [26] for review of empirical and theoretical evidences [27]). More specifically, the theoretical and modeling studies described above would predict a hyper-conformist response.

## Methods

### Study Site

The study took place in the ‘Living Links to Human Evolution’ Research Centre, a field station of the University of St. Andrews situated within the Royal Zoological Society of Scotland's Edinburgh Zoo (see http://www.living-links.org/and [25]). The Living Links Centre is dedicated to behavioral research on mixed species groups of brown capuchin monkeys (*Cebus apella*) and squirrel monkeys (*Saimiri sciureus*). The Centre is divided into East and West wings comprising two enclosures that mirror each other. Each wing of the building contains one large outdoor enclosure connected to two inside enclosures with a research room in between them. Visitors can watch what happens in the inside enclosures and in the research room by walking along a corridor that runs at the back of these rooms. Between the two wings of the building, visitors can view both outside enclosures from a large platform, or enter an enclosed section of corridor joining the two wings. This area, known as the ‘Science Exploration Zone’, contains signs and interactive computer games explaining the purpose of Centre and the research conducted there.

**Figure 2:**
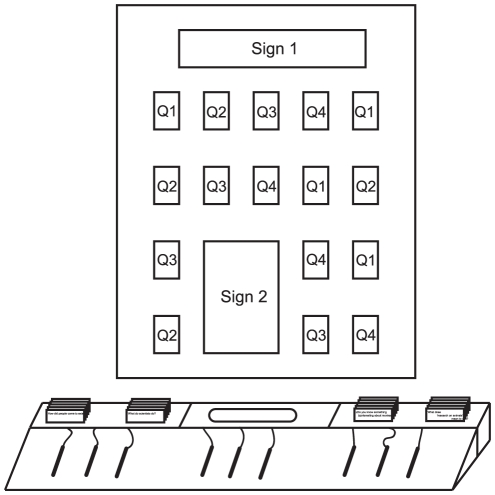
Schematic illustration of the drawing board and secured display panel. Q1 to Q4 refers to the questions described in the main text. Sign 1 reads: “Share your ideas! For people of all ages”. Sign 2 reads: “Win a Prize! Share your ideas… Just complete a card! Don't forget your age and email so we can tell you if you win! Prizes for adults (over 16) Prizes for children (below 16) Post your card here [arrow pointing towards the posting slot]”.

The present research took place in the ‘Science Exploration Zone’. In spring 2010 a new public engagement activity was introduced. A large wooden desk with colored pencils attached on cables was added to a large bench. Visitors were encouraged by two signs to answer questions about the Centre and its activity on A5 cards (see material below). One sign read “Share your ideas! For people of all ages”. and the other one read “Win a Prize! Share your ideas… Just complete a card! Don't forget your age and email so we can tell you if you win! Prizes for adults (over 16) Prizes for children (below 16) Post your card here” (see also Fig. 2). Participants could write or draw a response, or answer with a combination of text and drawing. To stimulate participation, a small prize was advertised and some examples were put on display above the board.

### Participants

Participants were visitors to Edinburgh Zoo and the Living Links Centre. To avoid interfering with the experiment itself, given its nature, we could not directly record data on visitors taking part in the activity, but demographic data were collected for the Centre and the Zoo. Demographic data show that there were 2500 visitors per day in the Zoo on average during June, July and August 2010: 14% on average were children under 3 years of age, 30% on average were children (aged 3 to 15 years of age) and 56% on average were adults (above 15 years of age). Mean group size visiting the Centre was around 3 (Mean+/−SD = 3.2+/−1.2; N = 94). Mean visit duration at the Centre was about 12 minutes (Mean+/−SD = 710+/−280 sec; N = 94). Informal observation suggested that children - sometimes with parents supervising - were most likely to participate. Large groups were unlikely to spend time on this activity.

### Materials

The general purpose of the activity was to ask visitors questions to assess their views, knowledge and understanding of the Centre in an entertaining way. Visitors could pick a card and draw and/or write an answer, working on a wooden desk (see Fig. 2). The desk was 122 cm wide, 48 cm deep, and 17 cm high at the back, with a slightly angled top to facilitate writing. Six to nine pencils of different colors were attached to the desk by thin cables. At the rear were two boxes in which A5 cards were presented, with a posting slot in the middle to post cards.

Visitors could answer each question on a black and white, double-sided A5 card. On one side a question was printed at the top with a black frame (12.5×15.5 cm) in which to respond. On the reverse, optional information was requested (name, age, gender and email address) along with the following text: “We will post the best responses on our website and on the wall at Living Links with the age and first name of the participant. If you provide your email address we can let you know if your picture has been selected. We will not use your email address for any other purpose.” We used the expression ‘best responses’ in order to emphasize the necessity to behave optimally because the theory predicts that a hyper-conformity arises when individuals are trying to behave in the most effective way [8], [19]. The expression ‘best responses’ also leaves somewhat ambiguous what ‘best’ may mean and therefore preserves the uncertainty of the task. However, insisting on the competitive aspect of the situation could potentially favor anti-conformity, the choice of a behavior opposite to what the majority is doing. In this case, we would expect a negative, and not a positive relationship between the percentage of text displayed and the proportion found in the answers (the non-shaded areas of Figure 1).

Above the desk, a display panel (120×90 cm) was used to pin up 16 answers and to give instructions (Fig. 2). Two text blocks explained the purpose of the drawing board to potential participants (Fig. 2). Between these text blocks were two lines of five cards each; two cards were additionally placed on the left, and four on the right side of the bottom block, respectively.

Before starting the experiment, we analyzed the cards that were collected as part of the normal functioning of the activity. At the time, four questions were asked of visitors:

Q1: What does ‘research on animals’ mean to you?

Q2: What do scientists do?

Q3: How did people come to exist?

Q4: Do you know something interesting about monkeys?

From the responses for each question we selected four with only writing and four with only drawing. This represented a total of 32 cards that could be used to manipulate the proportion of examples displayed on the wall. During the experiment, only these four questions and their associated 32 examples were used.

### Procedure

Seven sessions for each of five conditions (0, 25, 50, 75 and 100% ‘Text Displayed’) were completed between June and August 2010. Every session started with a selection of the appropriate number of drawing and writing examples pinned on the display panel. For instance, for the 25% ‘Text Displayed’ condition we selected three drawing cards and one writing card for each of the four questions. In order to keep the display homogeneous the position of each question was fixed throughout the experiment (as in Fig. 2). We also preserved a uniform distribution of drawing and writing by distributing examples evenly across the board.

When the display was ready, the experimenter refilled the drawing board with 10 blank answer cards for each of the four questions. Once all 40 cards had been used, the session was stopped and a new session could be started. Each session took between half a day and two days, depending on the number of visitors coming to the Zoo and their willingness to participate.

### Data Coding

We were interested in the change in the proportion of text and drawing in responses resulting from our manipulation of the proportion of text and drawing on display (% ‘Text Displayed’). We anticipated that conformity could be manifest in two different ways; the proportion of text could change within responses on each card, or the proportion of cards with only text or only drawings could change. We therefore asked two students, aged 21 and 30, blind to the purpose of the experiment and to the condition in which each card was realized (the cards were randomly ordered before coding), to evaluate all cards according to the following criteria (this is a verbatim copy of part of the written instructions given to these coders):

#### Text only

Text only is a card with only text written on it, any amount, from a single word to several paragraphs (‘smileys’ and other text associated characters are included). 1: belong to the category; 0: does not belong to the category.

#### Drawing only

Drawing only is a card with only drawings on it but name and age can be included. 1: belong to the category; 0: does not belong to the category.

#### Mainly text

Mainly text is a mixed card with text and drawing but with proper sentences not included in the drawing. Proper English sentences can be long ‘The monkey is eating an apple.’ or short ‘Watch!’ and express statements ‘I think we should go now.’, questions ‘What do you want?’, request ‘Could you come here?’, command ‘Don't do that!’, etc. These sentences should not explicitly be included in the drawing with arrows, text bubble or anything like that. 1: belong to the category; 0: does not belong to the category.

#### Mainly drawing

Mainly drawing is a mixed card with text and drawing but with no proper sentences or sentences included in the drawing as part of a legend, speech bubbles, etc. 1: belong to the category; 0: does not belong to the category.

#### Quality

The quality of the answer should not reflect your opinion on the question (whether or not you agree with the answer given) or the state of the card (foot prints, tears, etc) but rather the effort the participant has invested in answering the question. Try to consider this in terms of effort rather than the ability of the participant. Please rate the quality of the answer using the following scale. (0) Extremely poor, (1) Very poor, (2) Poor, (3) Good and (4) Very good, (5) Extremely good.

The first four criteria listed above were used to determine whether a given card was more of a drawing or of a text nature. Additionally, informal study of the cards revealed a large proportion of poor quality answers (scribbles, defaced cards, etc), so we asked the two coders to rate the ‘Quality’ of each card on the five point Likert scale described above in order to be able to exclude very poor quality cards from the analysis.

Finally, to reflect the two different ways in which conformity could affect the results we calculated two proportions for each session: % ‘Text Produced’ was the number of cards in Text only and Mainly text divided by the number of cards in all four categories; % ‘Text Produced only’ was the number of cards in ‘Text only’ divided by the number of cards in ‘Text only’ and ‘Drawing only’.

## Results

### Inter-coder Reliability

Inter coder reliability analysis was performed on the full dataset (1105 cards). Cohen's Kappa was 95% for ‘Text only’, 90% for ‘Drawing only’, involved in the calculation of % of ‘Text Produced only’, and 90% for both ‘Text only’ plus ‘Mainly text’, and ‘Drawing only’ plus ‘Mainly drawing’, involved in the calculation of % ‘Text Produced’. Given the very high inter coder reliability, we present the results obtained with data coded by the first coder only.

Across all conditions, 367 cards from 1105 (33%), rated as ‘Extremely poor’ or ‘Very poor’ by both coders, were excluded from the analysis. Table 1 summarizes the number of cards analyzed for each question and category.

**Table 1:** Frequency table of number of cards analyzed for each question and category.

	Question				Category				
% Text Displayed	Q4	Q3	Q1	Q2	Text only	Mainly text	Drawing only	Mainly drawing	Total
0	39	31	35	36	22	28	65	26	141
25	39	36	36	30	56	30	49	6	141
50	45	41	31	41	67	42	33	16	158
75	46	35	24	29	79	26	23	6	134
100	44	41	44	35	136	16	9	3	164
Total	213	184	170	171	360	142	179	57	738

### Effect of the Proportion of Text Displayed

The analysis of % ‘Text Produced’ and % ‘Text Produced only’ gave qualitatively similar results; we therefore report only the most comprehensive measure, % ‘Text Produced’.

**Figure 3:**
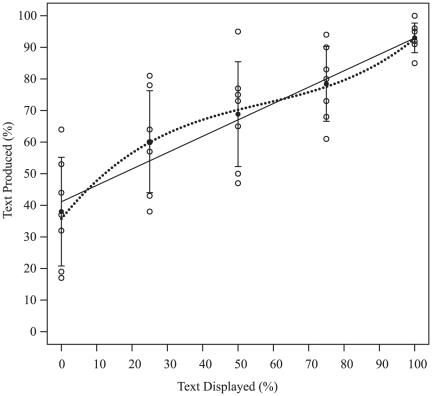
Influence of % ‘Text Displayed’ on % ‘Text Produced’. Circles represent proportion for each of the 7 sessions in the 5 conditions (summed across all questions asked). Disks represent the mean over the 7 sessions and error bars are+/−standard deviation for % ‘Text Produced’ in each of the five experimental conditions (0, 25, 50, 75 and 100% ‘Text Displayed’). The solid line represents the linear fit and the dotted line the cubic fit using the parameters in Table 3.

Firstly, we were interested in the preference of participants to draw or write, because the effect of conformity is more likely to be detected when both of the alternative behaviors are likely to occur with equal probabilities. If one behavior is much more likely than the other, changes in proportions may be masked by ceiling or floor effects (because proportions are contained within the [0; 1] interval). Therefore, when designing the experiment, we chose to use open questions to stimulate writing and provided blank cards and color pencils to stimulate drawing, hoping that these two effects would roughly balance each other. If the two behaviors, writing and drawing, were equally likely to occur, when the proportion of ‘Text Displayed’ is 50% the mean proportion of text found in the answers should also be 50%. It is also the case that if the two behaviors were equally likely, we could expect 50% text in a control condition in which no card would be on display. However, the absence of cards in this scenario makes any comparison with the other conditions in which there are examples on display quite remote. We think that comparing the 50% ‘Text Displayed’ with other conditions offers a more appropriate control. As illustrated in Figure 3, in the 50% ‘Text Displayed’ condition we found that on average % ‘Text Produced’ is significantly greater than 50% (Mean+/−SD = 69%+/−16.6, N = 7; two-tailed one sample t-test, t(6) = 3.006, p = 0.024), suggesting that there was a slight preference for writing over drawing. There was much scope however, for ‘Text Produced’ to increase or decrease without ceiling or floor effects. In only one session, in the 100% ‘Text Displayed’ condition, did % ‘Text Produced’ reach 100%; and it never fell to 0%.

The preference for writing is also confirmed by the fact that when there is no text on display, the proportion of text produced in the answers is significantly greater than 0 (Mean+/−SD = 38%+/−17.2, N = 7; t(6) = 5.844, p = 0.001). This bias for writing, inherent in the design of the experiment, has to be taken into account when comparing Figure 1, the theoretical case in which the two alternatives are perfectly equivalent, with Figure 3, in which there is a preference for writing (see below).

Next, we analyzed the main effect of ‘Text Displayed’ on ‘Text Produced’ using the statistical package R [28] to construct a binomial generalized linear model with an identity link. We found a highly significant effect of ‘Text Displayed’ on ‘Text Produced’ (Pearson Chi-square, d.f. = 1, Deviance = 126.09, p<0.001).

We were further interested in the nature of the conformist effect and in particular in the presence of a non-linear effect that would potentially reveal the presence of weak- or hyper-conformity. We fitted a linear and cubic model to our data (using the same generalized linear model procedure) and calculated the AICc (Corrected Akaike Information Criteria [29], [30]) and AICc weight for the two models (results are summarized in Table 2). If there is a weak- or hyper-conformist effect, the cubic model should fare better than the linear one.

**Table 2:** Comparison between the null, linear and cubic models.

Model	N	Log Likelihood	AICc	AICc weight
Cubic	4	−81.83	171.72	0.61
Linear	2	−84.29	172.60	0.39
Null	1	−147.48	296.97	0.00

The linear and cubic models fare better than the Null model of a constant ratio of Text over Drawing. Furthermore, there is a slight advantage of the cubic model over the linear one as can be seen by compaing the AICc weights.

As can be seen in Table 2, the difference between the linear and cubic model is small. There is a slight advantage for the cubic model over the linear one (AICc is used to identify which model provides the best fit to the data; the lower the AICc the better the model fit. The difference in AICc as well as the difference in weight gives an indication of the advantage of one model over another. The weight, in particular, can be understood as the relative likelihood of each model being the best model in the set. Here there is roughly a 60/40 split between the two models; therefore, there is a small advantage of the cubic model over the linear one). However, as can be seen in Table 3, the contribution of the cubic term is not significant, it only approaches significance. These results (see also Figure 3) therefore suggest that there *might* be a small nonlinear effect on top of the main, linear one ([30] recommend relying exclusively on AIC values when fitting models, not on the significance of the effect of single parameters.).

**Table 3:** Parameters for the linear and cubic models.

	Linear model		Cubic model			
Parameters	Intercept	Linear term	Intercept	Linear term	Square term	Cubic term
Estimate	4.11E-01	5.18E-01	3.57E-01	1.39	−1.98	1.16
Std. Error	3.00E-02	3.90E-02	4.00E-02	3.97E-01	9.83E-01	6.28E-01
z value	13.46	13.27	8.93	3.5	−2.02	1.85
p value	<0.001	<0.001	<0.001	<0.001	0.043	0.064

It is of theoretical interest whether any such effect is more consistent with a hyper-conformist effect rather than a weak one. Weak- versus hyper-conformity can be discriminated by the shape of the best fit: if it is S-shaped it corresponds to hyper-conformity and an inverse ‘S’ corresponds to weak-conformity (Figure 1). A simple visual inspection of Figure 3 reveals that the curve corresponds to weak-conformity and not to hyper-conformity (More precisely, using the results of the model described in Table 3, we find that the second derivative of the cubic function is positive between 0 and approximately 56.8% and negative afterwards).

To summarize, we found a positive and significant effect of ‘Text Displayed’ on ‘Text Produced’, showing a general positive relationship between the two variables and therefore, according to our definition, a conformist effect. When we investigated further the nature of that effect, we found a small advantage of the cubic model over the linear one, suggesting that there might be small nonlinear effect on top of the main linear one. This effect would correspond to a small weak-conformist effect and we therefore conclude that the main effect corresponds essentially to linear conformity, with potentially a small additional weak-conformist effect. We did not find evidence of hyper-conformity.

## Discussion

Using a real, everyday setting, in which visitors to an exhibit could write or draw on a card and post their answer for future display and enter a prize draw, we found a strong and significant effect of the manipulation of the proportion of text displayed on the proportion of text produced in the answers. Participants in this situation therefore showed clear evidence of conformity; they were more likely to write when there was a greater proportion of text displayed.

We were further interested in teasing apart the three kinds of conformity described in the Introduction (recall Figure 1). We found that the frequency of text over drawing had a strong effect on the behavior of participants, but this effect was essentially linear, with some evidence of an additional weak-conformist tendency rather than with hyper-conformity.

Theoretical modeling studies have shown that hyper-conformity can evolve to deal with environmental uncertainty. In an uncertain environment, hyper-conformist individuals may do better than linear or weak-conformist individuals because they are more likely to adopt the most frequent behavior; which is likely to be appropriate [8]. In line with this argument, experiments have focused on contexts in which participants are uncertain about the outcome of their behavior. The context of our experiment is also uncertain because participants were not aware of the criteria and procedures that determined what kind of answer was likely to be successful in getting displayed. In order to cope with this uncertainty, participants could look for additional information and use examples on display as a source of information. Nevertheless, participants failed to be hyper-conformist in our experiment.

Other factors, despite uncertainty, that can influence the strength of hyper-conformity have been less studied but two could potentially explain our results. First, the competitive aspect of the experiment might have favored distinctiveness rather than conformity. However, an advantage of our design in this respect is that distinctiveness regarding the means to answer is limited (there are only three possibilities, one can draw, write or do both to answer a question - and in three out of five conditions there were already both drawings and writings on display). Furthermore, if distinctiveness regarding writing or drawing had been important, we would have expected a negative relationship between what was displayed on the board and the response of the participants, which is the opposite of what we find. We conclude that the absence of hyper-conformity in the means to answer the questions is unlikely to be explained by any significant pressure for distinctiveness.

A second factor that could explain the absence of hyper-conformity might be a lack of motivation. According to this hypothesis, the stakes might have been too low to induce hyper-conformity in the participants. However, if motivation was low, we would expect participants not to pay attention to the display and this would have resulted in an absence of conformity (a straight horizontal line in Figure 3). To the contrary, participants in our experiment were clearly influenced by the cards on display. It remains an empirical question whether the shape and strength of conformity can be influenced by motivation. This represents an interesting unexplored avenue for future research that could potentially deepen our understanding of conformity.

We note, however, that our results and those of others [16], [22], [23], [31] show that participants tend not to be hyper-conformist in natural situations and that results in more controlled, but also more artificial settings do not provide compelling evidence that such a bias exists [10], [13], [18], [20], [21], [32]. At one extreme, lack of evidence for such a bias in these contexts could mean that it is actually rare or absent in humans [16] and thus that although hyper-conformity could theoretically be an important evolutionary force it has not in fact evolved, for reasons yet to be established. However, only a few experiments specifically addressing the existence of hyper-conformity in humans have been carried out so far, so it would be premature to settle on this conclusion at this juncture.

Another possibility to explain the lack of evidence for hyper-conformity is that experiments have focused on a very specific context. In social psychology two kinds of motivations underlying conformity have been recognized [33]. According to Campbell and Fairey [34]:

“Informational influence is based on the desire to be accurate; others' responses are used as a source of information about reality, and people conform because they believe that the others may be correct. Normative influence is based on the desire to maximize social outcomes. Even when people believe the others are wrong, they may conform in order to gain the rewards or avoid the punishments that such agreement and disagreement mediate.” p 458

Being conformist in an uncertain situation would correspond to informational conformity because others' behavior is used as a source of information about the situation. It could be that weak- and linear conformity are associated with informational conformity and hyper-conformity could be associated with normative conformity [9]. According to this hypothesis, future research on the existence of hyper-conformity might be well advised to focus on the normative context.

Finally, from an evolutionary point of view it is important to note that the combination of a slight preference for one of two options - writing text in our experiment – together with linear conformity produces convergence toward the more common option. Imagine we were to start a new experiment with only drawings on display for the first session and then, for each new session, with a random selection (among the not too low-quality cards) of the cards from the previous session. Based on our results, we can predict that the proportion of writings would progressively increase to reach roughly 80%. A slight tendency to perform one behavior over another (what is often referred to as a direct or content bias, [8]), combined with linear conformity, can therefore be amplified by cultural transmission and increase group homogeneity. Given such a process, the advantages of hyper-conformity over simple linear conformity are not so obvious. This issue deserves to be studied empirically in more substantial ways, extending to the variations in context discussed above.

In conclusion, there is a renewed interest in the study of conformity because theoretical models suggest that hyper-conformity could be a very important force in cultural evolution and recent experiments with animals indicate that conformity might not be uniquely human (see for instance [24], [35], [36], [37]). However, few experiments on humans have attempted to study the influence of the frequency of different information on behavior in a natural context.

The present experiment provides evidence clearly inconsistent with hyper-conformity in humans in a very natural context. By manipulating the proportion of writings vs. drawings on display, we were able to show linear or slightly weak-conformity but not hyper-conformity.

However, even if hyper-conformity is not present in informational contexts, it might still occur in more normative ones. Future experiments looking for evidence of hyper-conformity should therefore also explore more normative contexts.
